# Effect of storage temperature and time on erythrocyte sedimentation rate

**DOI:** 10.1186/s40001-022-00701-3

**Published:** 2022-05-28

**Authors:** Qi-Lei Hu, Zuo-Jie Li, Li Lin, Liang Zhang, Yin-Jiang Lv, Li-Feng Wu, Mei-Yun Chen

**Affiliations:** 1 Department of Clinical Laboratory, Linping Campus, The Second Affiliated Hospital of Zhejiang University School of Medicine, Hangzhou, 311100 Zhejiang China; 2 Quality Management Section, Linping Campus, The Second Affiliated Hospital of Zhejiang University School of Medicine, Hangzhou, 311100 Zhejiang China; 3 Department of Clinical Laboratory, The People’s Hospital of Cangnan Zhejiang, Cangnan County, 325800 Zhejiang China; 4 Department of Rehabilitation, Taizhou Hospital of Zhejiang Province, Taizhou, 31700 Zhejiang China; 5 Department of Clinical Laboratory, Xinchang County People’s Hospital, No. 117 Gushan Middle Road of Nanming Street, Xinchang County, Shaoxing, 312500 Zhejiang China

**Keywords:** Erythrocyte sedimentation rate, Westergren method, Room temperature, Cold storage, Storage time

## Abstract

**Objective:**

This paper explores the effect of blood sample storage temperature and time on the erythrocyte sedimentation rate (ESR) by using the Weiss method.

**Methods:**

Whole blood samples were collected from 80 patients and diluted 1:9 with sodium citrate solution. Each sample was split into two tubes. Using the Weiss method, ESR was tested within 1 h of collection, and one sample was placed at 4 °C and the other at room temperature (23 ± 2 °C). ESR was then measured at 2, 4, 6, 8, 12, and 24 h. The data were statistically analyzed with consideration for temperature and time.

**Results:**

ESR decreased gradually over 6 h at room temperature, but the results were not statistically significant. Similarly, there was no significant difference in the decline of ESR within 8 h at 4 °C. However, ESR results decreased significantly after the samples were stored at room temperature for more than 6 h or at 4 °C for more than 8 h. ESR reduction was lower in the samples stored at 4 °C than in those stored at room temperature over the same time period.

**Conclusion:**

Blood sample storage temperature and duration can affect the measurement of ESR using the Weiss method. ESR testing should be completed within 4 h of sample collection in clinical work.

## Introduction

Erythrocyte sedimentation rate (ESR) is a nonspecific sickness index, which is not diagnostic of any particular disease [1–3]. However, an elevated ESR may indicate the presence of inflammation, infection, rheumatologic disease, or neoplasm in patients who are unwell. A normal ESR has a strong negative predictive value for these conditions [4–6]. Moreover, in a recent study, ESR was shown to be a strong predictor of mortality from coronary heart disease and appears to be a marker for aggressive forms of the disease [7, 8]. Thus, ESR has a diagnostic value in some conditions and allows monitoring of therapeutic interventions in others [9, 10]. It also responds to surgical intervention. The ESR test measures the distance in millimeters that erythrocytes fall during a specified time period as a function of acute phase proteins and the cellular composition of blood. The International Council for Standardization in Haematology has recommended the original method described by Westergren as the gold standard, which determines the sedimentation of erythrocytes after one hour in a vertically mounted tube of defined length and bore size [11, 12]. It is a straightforward, inexpensive, and routine test to assess and monitor many diseases. However, there has been little research regarding the stability of ESR at varying times and temperatures.

## Materials and methods

### Study population

The study population consisted of 80 patients at Linping Campus, The Second Affiliated Hospital of Zhejiang University School of Medicine, China. There were 42 males with a median age of 59 years (range 37–80 years) and 38 females with a median age of 58 years (range 36–79 years). The patients were known to have an abnormal ESR and were visiting the laboratory for routine ESR monitoring when asked to participate in the study (Table 1). The experiments were performed in the laboratory, and ESR was determined using the Westergren method. The study was approved by the Committee on Research Ethics at the hospital, and all patients gave their informed consent for inclusion in the study.

**Table 1:** Patient characteristics in the study groups

Characteristics	Value
Gender [*n* (%)]	
Male	42 (52.5)
Female	38 (47.5)
Total	80 (100)
Age (years), (M [Q1, Q3])	
Male	59 (42.72)
Female	58 (40.71)
Total	59 (41.73)
Study group [*n* (%)]	
Tuberculosis	12 (15)
Acute appendicitis	20 (25)
Rheumatoid arthritis	28 (22.5)
Gastric cancer	14 (17.5)
Bronchopneumonia	16 (20)
Total	80 (100)

### ESR measurement using the Westergren method

The procedure was performed according to the manufacturer’s instructions, using the manufacturer’s vials, graduated tubes, leveling rack, and vertical tube holder. Briefly, blood samples of study patients were collected in ESR vacuum tubes. From these samples, 1.6 ml blood was pipetted into a different ESR vacuum tube containing 0.4 ml of 3.8% sodium citrate as an anticoagulant and inverted 10 times to mix. Next, a plastic tube with graduated markings (0–150 mm) was inserted through the pierceable stopper to the bottom of the vial. This unit was then placed vertically into a level holder. The system was self-zeroing in that the red blood cells automatically leveled to the 0 mm mark at the top of the tube. After 60 min, the ESR measurement was read as the millimeter mark where the erythrocyte–plasma interface appeared. Daily quality control methods were performed during the study.

The baseline ESR (mm/h) was measured in the serum by the Westergren method immediately following sample collection. Within minutes, each sample was then placed at a temperature of either 4 °C or 23 ± 2 °C for up to 24 h. The maximum permissible storage time was assessed by measuring the ESR after 0, 2, 4, 6, 8, 12, and 24 h.

### Statistical analysis

Statistical analysis was conducted using SPSS version 22.0 software. The baseline ESR was compared with subsequent ESR readings at varying temperatures and duration using the Wilcoxon signed-rank test for non-parametric data. A *P*-value of less than 0.05 was considered to be statistically significant.

## Results

**Fig. 1:**
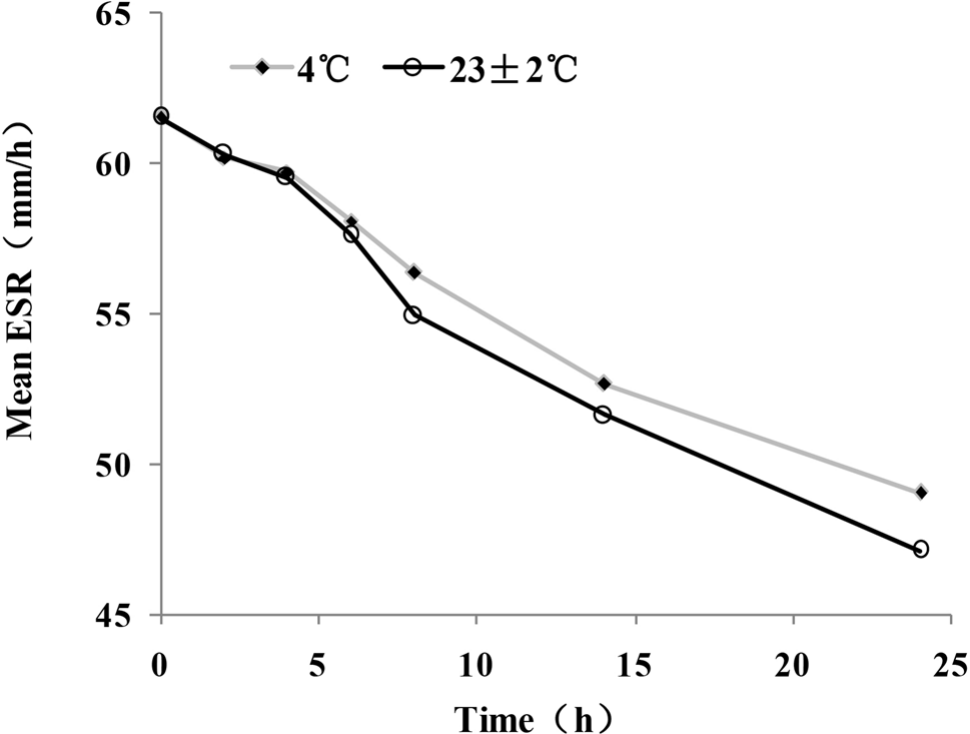
Effect of time on erythrocyte sedimentation rate at 4 °C and 23 ± 2 °C storage of blood samples

The baseline and follow-up ESR readings were evaluated for the 80 paired blood samples (Tables 2, 3, and Fig. 1). There was no significant effect on ESR in blood samples stored for 8 h at 4 °C (8.39% change in mean ESR). However, a significant decrease (14.38% decrease in mean ESR) was observed following storage for 12 h at 4 °C compared with the baseline ESR. In addition, there was no significant difference (6.32% decrease in mean ESR) between the baseline and the ESR reading following storage for 6 h at 23 ± 2 °C. However, there was a noticeable decrease in ESR (10.69% decrease in mean ESR) after storage for 8 h at 23 ± 2 °C. ESR decreased much less at 4 °C than at 23 ± 2 °C after 4, 6, 8, 12 and 24 h (0.31%, 0.8%, 2.3%, 1.71%, and 3.14% less decrease in mean ESR). There was no marked difference between the two temperatures after 2 h (0.18% more decrease in mean ESR).

**Table 2:** Effect of time on erythrocyte sedimentation rate at 4 °C storage of blood samples

	Baseline mean (SD)	Follow-up mean (SD)	Change in mean (%)	*P*^a^
ESR (2 h), mm/h	61.53 (17.70)	60.21 (16.94)	− 2.15	NS
	61 (29–105)	60 (29–103)		
ESR (4 h), mm/h	61.53 (17.70)	59.75 (19.67)	− 2.90	NS
	61 (29–105)	59 (28–101)		
ESR (6 h), mm/h	61.53 (17.70)	58.13 (15.98)	− 5.52	NS
	61 (29–105)	58 (27–98)		
ESR (8 h), mm/h	61.53 (17.70)	56.37 (18.37)	− 8.39	NS
	61 (29–105)	56 (25–95)		
ESR (12 h), mm/h	61.53 (17.70)	52.68 (16.88)	− 14.38	0.021
	61 (29–105)	52 (23–88)		
ESR (24 h), mm/h	61.53 (17.70)	49.05 (15.32)	− 20.28	0.007
	61 (29–105)	48 (21–83)		

^a^Wilcoxon signed-rank test

**Table 3:** Effect of time on erythrocyte sedimentation rate at 23 ± 2 °C (room temperature) storage of blood samples

	Baseline mean (SD) median (range)	Follow-up mean (SD) median (range)	Change in mean (%)	*P*^a^
ESR (2 h), mm/h	61.53 (17.70)	60.32 (16.50)	− 1.97	NS
	61 (29–105)	60 (29–102)		
ESR (4 h), mm/h	61.53 (17.70)	59.55 (16.67)	− 3.21	NS
	61 (29–105)	59 (28–101)		
ESR (6 h), mm/h	61.53 (17.70)	57.64 (15.86)	− 6.32	NS
	61 (29–105)	57 (27–99)		
ESR (8 h), mm/h	61.53 (17.70)	54.95 (17.38)	− 10.69	0.031
	61 (29–105)	54 (25–92)		
ESR (12 h), mm/h	61.53 (17.70)	51.63 (17.01)	− 16.09	0.008
	61 (29–105)	51 (23–86)		
ESR (24 h), mm/h	61.53 (17.70)	47.12 (14.72)	− 23.42	0.012
	61 (29–105)	46 (21–79)		

^a^Wilcoxon signed-rank test

## Discussion

Because the ESR results are at a low level in the normal range, the small influence of the storage time on the absolute value of ESR will cause the big change of correlation, so this study chooses high ESR to eliminate this influence. Our data suggest that the measurement of ESR is dependent on the duration and temperature at which samples are stored. ESR was not affected by storage for 8 h at 4 °C or for 6 h at 23 ± 2 °C. Compared with fresh blood samples, ESR values in the 6 h stored samples were lower, but did not reach statistical significance. The reduction in ESR value was smaller in samples stored at 4 °C than in those stored at room temperature. A significant decrease in ESR was observed when samples were stored for 12 h at 4 °C or for 8 h at 23 ± 2 °C. Therefore, when at least 12 h of storage is required, we suggest that samples should be kept at 4 °C, with the caveat that ESR may start to decrease from 8 h onwards. Our results are important to consider for studies investigating ESR or whenever ESR cannot be measured proximate to the time that samples are drawn. We suggest that samples can be stored at room temperature for up to 4 h, after which they should be stored at 4 °C.

In recent years, a number of laboratory apparatus fitted with alternative techniques for ESR measurement have been developed, and a new instrument, Test 1 (SIRE Analytical Systems), has been marketed. However, globally, many community service centers and rural hospitals with basic infrastructure cannot afford the cost of advanced equipment. In addition, sample testing conditions vary considerably between such establishments. Therefore, knowledge of the stability of ESR at varying times and temperatures is important.

Our findings concerning the decreased stability of ESR at 4 °C and 23 ± 2 °C concur with earlier studies. However, previously, the maximum duration of ESR stability was reported as 4 h [13]. The reason for this variation in time is unknown, but it may, in part, be accounted for by differences in disease pathology. If samples are stored at room temperature, ESR tests should be conducted within 6 h. ESR stability degrades over time due to changes in the shape of the red blood cells, which become spherical. These spherical cells are difficult to aggregate, affecting rouleaux formation. In addition, the interaction of charges between the membrane surface of the red blood cells and the plasma proteins is modified. If the specimens are stored in the refrigerator, they must be tested within 8 h, and prior to testing, samples should be brought to room temperature as rapidly as possible. Otherwise, ESR will be reduced due to the increase in plasma viscosity at cooler temperatures.

The results of this study show that under conditions of 4 °C and 23 ± 2 °C, ESR determination results gradually decrease with prolonged sample placement time, which is different from the research results of Yu et al. [14]. In addition, ESR stability in their study was maintained for only 2 h, whereas in our study, it was 8 h and 6 h for 4 °C and 23 ± 2 °C. The possible reason for this is that the detection methods and sources of the two samples are different. ESR measurements published by Jiang Fei using ethylenediamine tetraacetic acid anticoagulant blood in the TEST 1 m over different time periods are essentially consistent with this study at 4 °C but exhibit greater changes at room temperature [15]. Again, this may be due to differences in the detection method.

This study is not without its limitations. The outcomes are based on small sample sizes, and the binding affinity results are established on single ESR measurements at the baseline and not on repeat ESR measurements after storage. In addition, our study consists of samples taken from patients with different diseases, the pathology of which may cause variations within the measurements. Regardless of these issues, the results of this study clearly suggest that for best results in a clinical research setting, ESR should be measured as soon as possible after sampling, but certainly within 6 h when blood samples are stored at room temperature. Further studies are necessary to confirm whether storage exceeding 8 h at 4 °C can be justified.

## Data Availability

We declared that materials described in the manuscript, including all relevant raw data, will be freely available to any scientist wishing to use them for non-commercial purposes, without breaching participant confidentiality.
